# Protocol for the “Implementation, adoption, and utility of family history in diverse care settings” study

**DOI:** 10.1186/s13012-015-0352-8

**Published:** 2015-11-24

**Authors:** R. Ryanne Wu, Rachel A. Myers, Catherine A. McCarty, David Dimmock, Michael Farrell, Deanna Cross, Troy D. Chinevere, Geoffrey S. Ginsburg, Lori A. Orlando, for the Family Health History Network

**Affiliations:** Duke Center for Applied Genomics & Precision Medicine and Duke Department of Medicine, Duke University, 411 West Chapel Hill Street, Ste. 500, Durham, NC 27705 USA; Duke Center for Applied Genomics & Precision Medicine, Duke University, Durham, NC USA; Essentia Institute of Rural Health, Duluth, MN USA; Human and Molecular Genetics Center, Medical College of Wisconsin, Milwaukee, WI USA; Center for Urban Population Health, Aurora University of Wisconsin, Milwaukee, WI USA; Department of Molecular and Medical Genetics, University of North Texas, Fort Worth, TX USA; Clinical Investigations Facility, David Grant Medical Center, U.S. Air Force, Travis, CA USA; Duke Center for Applied Genomics & Precision Medicine and Duke Department of Medicine and Pathology, Duke University, Durham, NC USA; Duke Center for Applied Genomics & Precision Medicine and Duke Department of Medicine, Duke University, Durham, NC USA

**Keywords:** Risk stratification, Prevention, Primary care, Family health history

## Abstract

**Background:**

Risk assessment with a thorough family health history is recommended by numerous organizations and is now a required component of the annual physical for Medicare beneficiaries under the Affordable Care Act. However, there are several barriers to incorporating robust risk assessments into routine care. MeTree, a web-based patient-facing health risk assessment tool, was developed with the aim of overcoming these barriers. In order to better understand what factors will be instrumental for broader adoption of risk assessment programs like MeTree in clinical settings, we obtained funding to perform a type III hybrid implementation-effectiveness study in primary care clinics at five diverse healthcare systems. Here, we describe the study’s protocol.

**Methods/design:**

MeTree collects personal medical information and a three-generation family health history from patients on 98 conditions. Using algorithms built entirely from current clinical guidelines, it provides clinical decision support to providers and patients on 30 conditions. All adult patients with an upcoming well-visit appointment at one of the 20 intervention clinics are eligible to participate. Patient-oriented risk reports are provided in real time. Provider-oriented risk reports are uploaded to the electronic medical record for review at the time of the appointment. Implementation outcomes are enrollment rate of clinics, providers, and patients (enrolled vs approached) and their representativeness compared to the underlying population. Primary effectiveness outcomes are the percent of participants newly identified as being at increased risk for one of the clinical decision support conditions and the percent with appropriate risk-based screening. Secondary outcomes include percent change in those meeting goals for a healthy lifestyle (diet, exercise, and smoking). Outcomes are measured through electronic medical record data abstraction, patient surveys, and surveys/qualitative interviews of clinical staff.

**Discussion:**

This study evaluates factors that are critical to successful implementation of a web-based risk assessment tool into routine clinical care in a variety of healthcare settings. The result will identify resource needs and potential barriers and solutions to implementation in each setting as well as an understanding potential effectiveness.

**Trial registration:**

NCT01956773

## Background

Failure to assess risk for common chronic diseases before they develop increases the likelihood that primary care patients will be faced with premature morbidity and mortality. Risk assessments integrate data from multiple sources including laboratory, biometric, genetic, environmental, and behavioral. Though the type of data synthesized for any one disease depends upon the risk algorithm, many rely heavily upon a detailed family health history (FHH), and in some cases such as Lynch syndrome, hemochromatosis, cystic fibrosis, and hereditary arrhythmias, FHH is the only data source [1–6]. Morbidity and mortality reductions are achieved by linking risk assessment results to evidence-based risk management guidelines which can both improve outcomes and more efficiently allocate medical resources in comparison to “one size fits all” medicine by, for example, encouraging appropriate timing and frequency of colorectal cancer surveillance [7], appropriate timing and method of breast cancer surveillance [8–10], breast cancer chemoprevention [11, 12], and cancer genetic counseling [13–16]. Given these benefits, risk assessment with a thorough FHH is recommended by numerous medical organizations, including the Centers for Disease Control and Prevention [17], U.S. Office of the Surgeon General [18], American Heart Association [4], and American Society of Clinical Oncology [1].

Implementing FHH-based risk assessment and management guidelines into practice is hindered by system-, clinician-, and patient-level barriers. System barriers include limited time available to record a thorough FHH [19–22] and lack of data standardization. Clinician barriers include limited awareness of the necessary data elements for risk stratification (e.g., age of onset) and limited training in how to synthesize FHH data into a risk management plan [23–26]. Patient barriers include limited knowledge about their FHH, the essential elements of FHH to provide, and the benefits of risk management [17, 24]. Health IT tools that collect patient-entered FHH and provide risk-based clinical decision support (CDS) have overcome some of these barriers. Evaluation of these tools has shown that they improve collection and documentation of high-quality FHH in 46–78 % of patient encounters [27–29] without impeding primary care clinic operations. Further, these tools have demonstrated the potential for high clinical utility by successfully identifying individuals who were either unaware of or not adherent to risk-based management [28, 30–32] and improving adherence to cancer screening [17, 33] and lifestyle change recommendations [34, 35].

In 2004, the Genomedical Connection, a collaboration by Duke University, University of North Carolina at Greensboro, and Cone Health, developed the genomic medicine model to help integrate personalized medicine into North Carolina primary care practices [36]. One key component of this model was the development and implementation of MeTree, a web-based patient-facing FHH-driven risk assessment and clinical decision support tool with integrated just-in-time education [37]. The initial version of MeTree, which collected data on 48 medical conditions and generated clinical decision support on five diseases (hereditary cancer syndromes, breast cancer, ovarian cancer, colon cancer, and thrombosis), was successfully piloted in three Cone Health community-based primary care clinics (two intervention sites and one control). These results included broad-based support from both patients and providers for its ease of use [38], improved identification of increased risk primary care patients [38, 39], high quality of FHH collection [40, 41], and increased alignment of patient care with risk management guidelines (paper in review).

These encouraging results led to grant funding from NHGRI and NCI as part of the Implementing Genomics in Practice (IGNITE) network (http://www.ignite-genomics.org) to optimize MeTree and evaluate its uptake and impact across a variety of diverse real world settings. Optimization and expansion of MeTree have been completed and include a tablet friendly user interface, help text linked to MedlinePlus Connect, incorporation of American Health Information Community’s requirements for FHH collection [42], full HL7 standards compatibility (www.hl7.org), data linked to ICD-9 and SNOMED codes for interoperability, data collection for 90 conditions, clinical decision support for 30 conditions (breast cancer, colon cancer, lung cancer, ovarian cancer, hereditary cancer syndrome, hereditary cardiovascular diseases, connective tissue diseases, hereditary liver diseases, abdominal aortic aneurysm, type 2 diabetes, coronary artery disease, and ischemic stroke), and the addition of a Spanish version. In this paper, we describe the pragmatic cluster controlled implementation-effectiveness hybrid type III trial designed to evaluate the implementation uptake and clinical utility of MeTree in five diverse healthcare systems across the USA.

## Methods/design

### Models

In order to effectively and efficiently integrate the FHH intervention into clinical practices, we employed an implementation sciences approach based upon the reach, efficacy, adoption, implementation, and maintenance (RE-AIM) model [43] and the Weiner organizational model of innovation implementation [44]. This approach is the key to understanding the optimal adaptations necessary for maximizing the impact of risk assessment programs (here represented by MeTree) across a diversity of settings—a critical component for facilitating widespread adoption.

The RE-AIM framework assesses an intervention’s potential to broadly improve population health, and the likelihood it will be translated into clinical practice. The model measures the following: *Reach* (the number, percent, and representativeness of the eligible intervention population), *Effectiveness*, *Adoption* (the number, percent, and representativeness of the participating intervention sites), *Implementation* [the extent of intervention delivery as intended (integrity) and frequency of use (exposure)], and *Maintenance* [43].

**Fig. 1:**
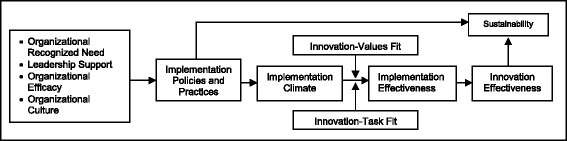
Weiner’s organizational model of innovation implementation

The adapted Weiner organizational model of innovation implementation (Fig. 1) builds upon the RE-AIM model by providing explanatory characteristics for the RE-AIM’s measures. Within the Weiner model, *Implementation Policies and Practices* are organizational strategies for using an innovation and the actions that follow. Examples are education and training, recognition and reward, communication and coordination, and time to experiment [45]. *Implementation Climate* is employees’ perception of the organization’s expectation for innovation use [46]. *Innovation-Task Fit* is compatibility with task demands, processes, and organizational capabilities. *Innovation-Values Fit* is compatibility with users’ values [46–49]. *Implementation Effectiveness* is the consistency and quality of innovation use [46, 50–52]. *Innovation Effectiveness* is the organizational benefits that accrue from innovation use (i.e., improved clinical care) [46, 48].

### Overview of study design

This clinical trial includes five national healthcare systems with distinct missions and operational profiles: Duke University Medical Center, Medical College of Wisconsin (MCW), Essentia Institute of Rural Health (EIRH), University of North Texas (UNT), and David Grant U.S. Air Force Medical Center. Duke and MCW are both academic health centers but each enrolled clinics that represent different populations: highly educated middle class, inner city, blue collar, and suburban. The clinics at EIRH represent rural populations, David Grant military populations, and UNT migrant Hispanic populations.

To understand the characteristics of each of these settings and how they impact uptake and clinical utility, we developed a pragmatic cluster hybrid implementation-effectiveness type III protocol with three phases (Table 1): pre-implementation assessments, implementation with strategic adaptations, and post-implementation assessments [53]. Hybrid study designs provide a structure for the complex process of collecting two entirely different types of information: implementation (how well the intervention is taken up by the clinical sites) and effectiveness (the clinical impact of the intervention). The choice between type I, II, or III designs depends upon the amount of underlying effectiveness data, in the case of type III studies, the effectiveness data is extensive enough that implementation is the primary outcome and effectiveness the secondary outcome [53].

**Table 1:** Hybrid implementation-effectiveness design elements^a^

Pre-implementation	Implementation	Post-implementation
• Identify current practice patterns	• Assess implementation *integrity* (used as intended)	• Assess acceptance and satisfaction for stakeholders
• Identify barriers and facilitators	• Assess implementation *exposure* (used at intervention sites)	• Assess clinical impact for all stakeholders
• Assess feasibility	• Identify explanations and solutions for low integrity or intensity	• Adapt and finalize implementation strategy
• Establish implementation plan	• Modify implementation plan	• Assess impact of final implementation strategy

^a^Adapted from [64]

### Recruitment, enrollment, and sample size

Primary care clinics within each of the five healthcare systems represent five states, 20 clinics, 79 providers, and ~45,500 unique patients per year from a variety of sociodemographic backgrounds (Table 2). Enrolled clinics were matched by sociodemographic factors to a representative clinic to serve as a control for the health system. Enrollment occurred in a stepped process with an initial implementation in one to four clinics. After 3–4 months, the remainder of the clinics began enrolling. The control clinics will convert to intervention clinics as part of the delayed roll out and will begin enrolling 1 year after study start date. Data from the control clinics will be used to account for temporal trends in clinical care.

**Table 2:** Clinical site demographics

	Duke	MCW	Essentia	UNT	Air force
State(s)	NC	WI	MN, WI, ND, ID	TX	CA
Setting	Academic	Urban	Rural	Migrant	Military
Female	61 %	51 %	54 %	61 %	19 %
Caucasian	59 %	77 %	99 %	46 %	73 %
Medicare/medicaid	27 %	NA	26 %	70 %	0 %
Uninsured	6 %	NA	8 %	1 %	0 N
Enrolled clinics	7	5	2	3	3
Waitlisted clinics	1	1	6	2	1

#### Providers

Since provider decision-making is integral to study outcomes, providers in the participating clinics are enrolled in the study. Provider-participants are recruited through in-person clinic meetings and individual email communication. A clinical champion at each clinic is identified to help facilitate implementation. Educational modules for providers were developed, including a website (http://dukepersonalizedmedicine.org/disease-risk-and-diagnosis/risk-assessments/family-history), a one-page summary of benefits and activities, and two webinars.

#### Patients

Patients of enrolled providers who have an upcoming well-visit appointment and meet inclusion/exclusion criteria (see below) are sent invitations (via mail or email) and educational materials 3 weeks prior to their appointment. Interested participants are enrolled into an entirely electronic protocol (see below) by a central coordinator. We anticipate enrolling *3000 patient-participants at a minimum* (to achieve significance for effectiveness measures), but as an observational study, we will continue to enroll as many as are interested in order to maximize our ability to assess differences across settings, populations, and sociodemographic factors. To reach this goal, we need to enroll ~157 patient-participants from each intervention clinic. Assuming a 10 % enrollment rate, we anticipate being able to enroll 4500 patient-participants.

#### Patient inclusion/exclusion criteria

Patients must be over the age of 18, English or Spanish speaking, and have an enrolled provider to be eligible. Since this proposal focuses upon prevention and not disease management strategies, those with one of the CDS study disease (e.g., breast cancer) will not be excluded from enrollment but will be excluded from analyses relevant to that disease.

### Electronic protocol and participant flow

**Fig. 2:**
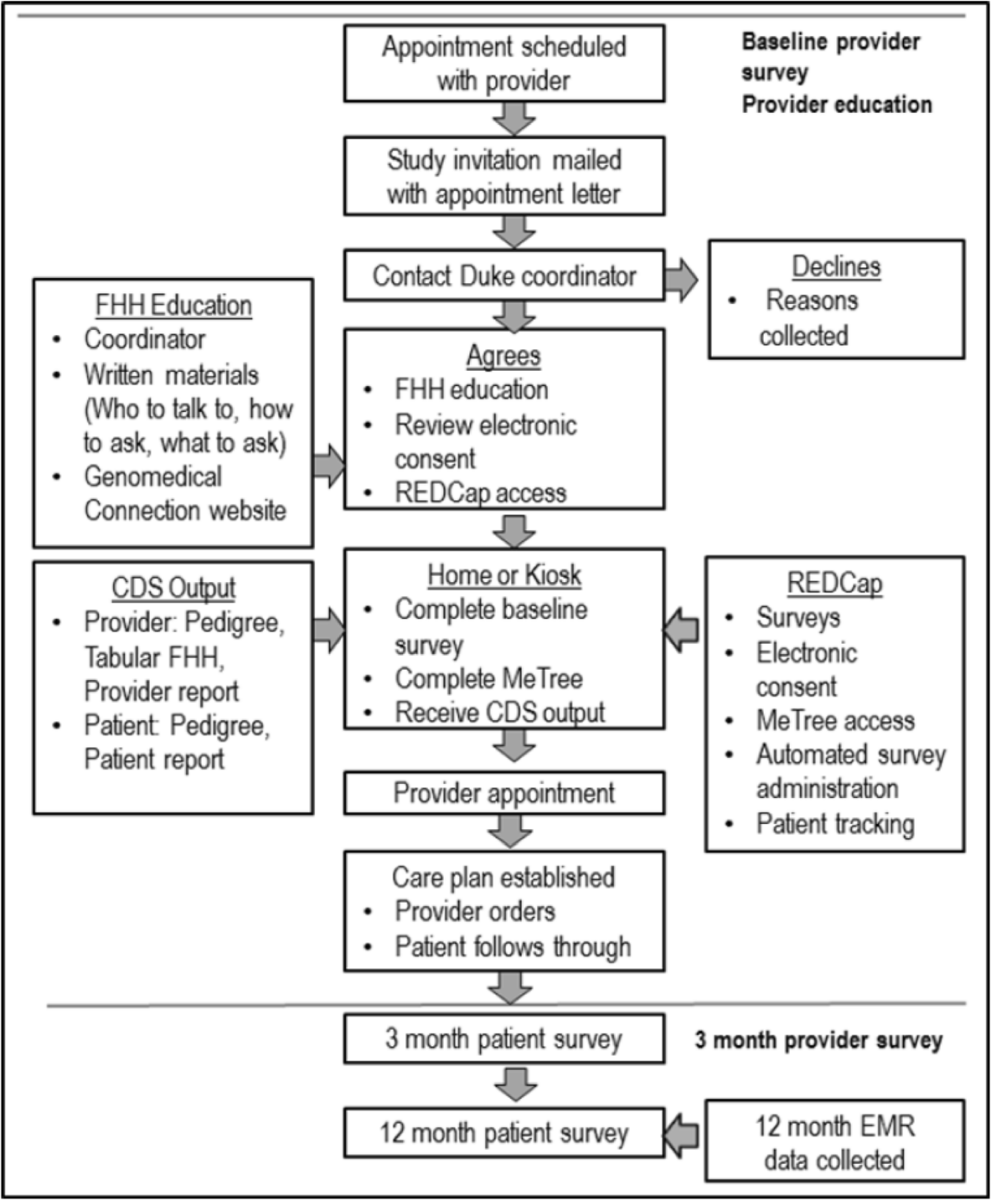
Study flow

Interested patients contact the study coordinator, either by phone or an electronic link embedded in the email invitation, to create an account in study system. At this point, the remainder of the study flow is entirely electronic. When they log-in to their account, they are emailed a link to an electronic consent. After consenting, they are emailed a link to complete a web-based baseline survey (Table 3). Upon completing the survey, they are emailed a secure link to access MeTree. They may log-in and out as often as they need to complete data entry. The patient-participant is required to complete MeTree 2 days prior to their appointment in order to upload the provider report to the medical record. At 3 and 12 months post-appointment, patients are sent an electronic survey to complete. In addition, an EMR data query for measures relevant to risk management and results will be performed at 12 months (Fig. 2).

**Table 3:** Domains of patient- and physician-oriented outcomes by data source

		Data source				
	MeTree	Patient surveys	Provider survey	Clinic staff survey (ORIC)	Clinic staff interview	EHR data pull
Emotional						
Satisfaction		●	●	●	●	
Barriers to model use		●	●			
Activation		●				
Quality of clinical encounter		●	●			
SF-12 (quality of life)		●				
Patient activation		●				
Knowledge			●			
Concur with/quality of CDS			●			
ORIC				●		
Implementation climate				●	●	
Behavioral						
Medication adherence		●				
Lifestyle		●				
Rec uptake		●	●			●
Discussion of risk/prevention			●			
Work flow/processes					●	
Implementation policies/practices				●	●	
Intervention values and task fit				●	●	
Biological						
Demographics	●					
FHH	●					
FHH documentation/ counseling			●			
% completion of MeTree	●					
Time to complete MeTree	●					
Clinical						
Laboratory data	●					●
Screening completed						●
Complications						●
Vital signs and weight	●					●
# medications						●
Referrals made						●
% high-risk patients	●					
% with risk-based screening	●					●
% with screening completed						●
% with disease at goal						●
Visit length/wait times			●			
Financial						
Socioeconomic status		●				
Medication costs						●
Office/ER visits/hospitalizations						●
Impact on family members		●

### Patient education and support

MeTree incorporates embedded FHH education on why FHH is important for their health, how to use the program, how to collect FHH from family members, what to ask about, and what information to gather about their own health history. A downloadable worksheet facilitates data collection of the key FHH components. Participants will collect and enter their own personal information and FHH into MeTree from a personal computer, mobile device, or a dedicated clinic kiosk. Within MeTree .api links to MedlinePlus Connect allow the display of low literacy content for a disease when the cursor hovers over the name. Participants are given a support email address and phone number to contact for assistance if needed.

### Delivery of results

Once patient-participants complete MeTree, the patient report is available in real time to print or save. A provider report including disease-oriented CDS based on current clinical guidelines (e.g., USPSTF, NCCN), a pedigree, and a tabular FHH is simultaneously generated and uploaded to the (E)MR. The provider-participant is then alerted to report availability. At the appointment, the clinical encounter proceeds as usual (Fig. 2).

### Study phases

#### Pre-implementation

Assessments during the pre-implementation phase were based on the adapted Weiner organizational model of innovation implementation. Mixed methods were used to assess characteristics related to the organizational environment, providers, and the patient population being served, in addition to perceived barriers/facilitators, potential adaptations, pros/cons of each intervention aspect (FHH collection, education, CDS output, CDS delivery, model integration, etc.), IT use, and comfort with FHH risk stratification (Table 1). A representative sampling of providers and staff was interviewed over the phone by a dedicated interviewer using a separate question guide for each position (nursing, clerk, provider, etc.). All providers and staff members at enrolled clinics were also invited to complete the organizational readiness for implementing change (ORIC), a validated survey instrument based on Weiner’s model [54].

#### Implementation and post-implementation

During implementation, progress-focused formative evaluations and summative quantitative measures assess characteristics related to implementation (implementation effectiveness, innovation effectiveness, sustainability/maintenance). Results are used to understand barriers arising during implementation, adapt the implementation to overcome those barriers, and identify critical elements required for the success of the risk assessment intervention in each environment. Providers and clinic staff are interviewed informally in an ongoing manner throughout the study and formally at 6 months post-enrollment. Providers are interviewed about satisfaction, unexpected barriers, impact on clinic processes and appointment quality, whether CDS was helpful in decision making, how patients reacted, and what would be necessary to establish the intervention as part of their routine clinical care. Nurses are interviewed about impact on workflow, patient questions, and how their role in the clinic may have changed.

### Study measures and outcomes

Quantitative data are obtained in three areas: (1) surveys of provider- and patient-participants around the uptake and acceptance of the intervention; (2) clinical effectiveness measures; and (3) patient-centered measures related to the clinical, behavioral, and emotional domains.

Given the natural tension between implementation measures, which require considerable input from participants, and pragmatic trials, which assume a hands-off observational intervention, we have devised measures across domains and stakeholders that meet both goals. Note that since the length of the study limits the ability to assess hard clinical outcomes such as reduction in cardiac events or incident cancer, we will use Healthcare Effectiveness Data and Information Set measures as intermediate clinical effectiveness measures for the CDS conditions. Measures are presented in Tables 3 and 4. Implementation outcomes are clinic, provider and patient adoption (enrollment rates), and representativeness to the underlying population. Primary effectiveness outcomes are percent newly identified high-risk individuals and percent with appropriate risk-based screening. Secondary outcomes include percent change in those meeting goals for a healthy lifestyle (diet, exercise, and smoking).

**Table 4:** RE-AIM implementation outcomes and measures

Outcomes	Measure	Source
Model reach	Representativeness of patient population to general population	Recruitment data (# enrolling/# invited); SES and demographics compared to overall population; compare across clinical settings and institutions
Effectiveness	see Domains of Measures and Outcomes Table	
Model adoption	Representativeness of clinics agreeing to participate	Recruitment data on clinic settings and characteristics as compared to general clinic settings at the institution; % of providers opting out and their characteristics compared to overall provider population in the clinics; formative evaluations on reasons for opting out
Implementation integrity	% time intervention used as intended	Formative evaluations, study coordinator tracking patient through steps in the model (ex. MeTree log-in vs completion), adaptations to the model, patient and provider FAQs derived during implementation, % time providers review CDS output
Implementation exposure	% time intervention used	Formative evaluations, study coordinator tracking patient through steps of the model
Maintenance and sustainability	Cost to implement	• EHR administrative data for utilization
	Cost/effectiveness	• Formative evaluations (clinic resource needs, successful elements for each setting, factors association with long-term adoption or not),
		• % adoption at study end
		• costs/disease prevented, early stage detected, or visits avoided

### Data analysis

Sample size calculations were preformed analytically using R. For each of the five behavior changes of interest, the baseline rates were obtained from the CDC’s Behavioral Risk Factor Surveillance Data [55] and are as follows: breast cancer screening—70 %, colon cancer screening—55 %, smoking—43 %, healthy diet—24 %, and controlled LDL—78 %. We analytically determined the number of patients required to detect a 5, 6, 7, 8, 9, and 10 % increase or decrease from the baseline rates in a one-sample test of proportions with a significance level of 0.05 and 80 % power. To account for within-clinic correlations in patient behavior, we estimated the variance inflation factor (VIF) assuming an intraclass correlation of 15 % [cite PMID: 22585888] with 20 intervention clinics for each behavior of interest. After accounting for the within-clinic correlation, 20 % attrition, a ~3:1 female to male ratio, and multiple behaviors/comparisons, approximately 2000 patients are needed to detect 10 % changes and 20,000 are needed to detect 5 % changes.

*Pre-implementation and Implementation qualitative and quantitative data* from physician and staff interviews and surveys will be reviewed by the study PIs and key personnel for themes to guide the development and adaptation of the implementation strategy. Particular attention will be paid to identifying potential barriers and facilitators that will lead to an implementation plan, which minimizes changes to workflow and staff duties. Implementation phase data, such as experience with the model, satisfaction, and impact on clinic workflow will be analyzed continuously and used to inform model adaptation until the time when optimization has occurred. At that point, frequency of provider data collection and analysis of both the patient and provider related data will decrease. *The final result will be an “implementation template” for each general setting*.

*Effectiveness data* will be summarized with descriptive statistics and plots. Generalized linear ordinal regression models (GLO) (the function ordglm from the R statistics package) will fit ordinal survey outcomes to the continuous outcome variables. Associations will be considered significant when the regression coefficient is not zero; a false discovery rate of 5 % will be used to correct for multiple comparisons. Multivariate analysis will control for clinic and provider. A *p* value of <0.1 in stepwise regression will identify significant factors such as demographics, intent to change, and their interactions. The analyses for the multiple outcomes will follow the same procedure as the survey outcomes but using a logistic regression model that includes the seven covariate factors (see sample size section). Although the study is implemented at the level of the clinical practice, the likelihood of clustering is low given that all participants undergo the intervention and the intervention is aimed at both the patient and the provider; however, to address the possibility of clustering, we will calculate a design effect [56]; if it is 1, we will use standard tests and generalized linear mixed models with clinic and state as random effects, if not, we will adjust the confidence intervals using a conditional logistic regression [57]. Effect size bias is extremely unlikely in this non-randomized study as all individuals receive the intervention, preventing the imbalance in treatment assignment that can lead to inaccurate point estimates [57].

*RE-AIM data w*ill be analyzed as in Table 4.

## Discussion

Risk stratification is an essential first step in mitigating risk and improving prevention efforts both on an individual and a population level. FHH is a critical component of that risk stratification and the most valuable and comprehensive “genetic test” we have available today. While patients and clinicians acknowledge the value of risk stratification and FHH [58, 59], there remain significant barriers to collection and utilization within clinical practice under current patterns of care [24, 25, 60, 61]. Development and use of electronic tools for collection and analysis of risk information has the potential to address many of these barriers [27–29] and to improve clinical care [29, 62, 63]. Yet at the same time, introducing technology into the clinical setting can present its own set of obstacles that must be evaluated and addressed.

This trial seeks to evaluate both the process of implementation of a web-based FHH platform into diverse clinical settings and its clinical effectiveness across those settings. Implementation outcomes will be measured using the RE-AIM framework. Patient-, provider-, system-level barriers and facilitators to implementation will be assessed through ongoing surveys of all participants and interviews of representative stakeholders throughout pre-implementation, implementation, and post-implementation phases of the study. Maintenance and sustainability will be measured by development of a CEA model to assess societal and institutional impact of such an intervention using prospectively collected data from the trial when possible. This will allow for evaluation of the potential impact of MeTree within different clinical settings and across the US population as a whole. Clinical effectiveness will be measured through (E)MR data pulls at the end of the study to assess provider and patient clinical activity as a result of the MeTree intervention as well as health behavior surveys of patient-participants pre- and post-intervention.

While application of technology within healthcare presents new challenges, it also provides opportunities to improve the care of patients and their families. This is particularly true when considering risk assessments which are best applied systematically with the most up-to-date and accurate data possible, something that has not been achievable in current practice. When applied thoughtfully and methodically, great benefit can be seen for patients and providers.

### Trial status

Providers and patient participants at Duke began enrolling April 2014, Essentia began enrolling May 2014, MCW began in October 2014, UNT began in July 2015 and David Grant began in October 2015.

## Abbreviations

CEA: cost-effectiveness analysis
CDS: clinical decision support
EIRH: Essentia Institute of Rural Health
(E)MR: electronic medical record
FHH: family health history
IGNITE: Implementing Genomics in Practice
MCW: Medical College of Wisconsin
ORIC: organizational readiness to Implement Change
UNT: University of North Texas
